# Rapamycin Eyedrops Increased CD4^+^Foxp3^+^ Cells and Prevented Goblet Cell Loss in the Aged Ocular Surface

**DOI:** 10.3390/ijms21238890

**Published:** 2020-11-24

**Authors:** Claudia M. Trujillo-Vargas, Shallu Kutlehria, Humberto Hernandez, Rodrigo G. de Souza, Andrea Lee, Zhiyuan Yu, Stephen C. Pflugfelder, Mandip Singh, Cintia S. de Paiva

**Affiliations:** 1 Grupo de Inmunodeficiencias Primarias, Facultad de Medicina, Universidad de Antioquia, UdeA, Medellín 050010, Colombia; cmvargas@bcm.edu; 2 Ocular Surface Center, Department of Ophthalmology, Cullen Eye Institute, Baylor College of Medicine, Houston, TX 77030, USA; hernandezh7@uhv.edu (H.H.); rodrigoguimaraes.rgs@gmail.com (R.G.d.S.); zhiyuan.yu@bcm.edu (Z.Y.); stevenp@bcm.edu (S.C.P.); 3 College of Pharmacy and Pharmaceutical Sciences, Florida A&M University, Tallahassee, FL 32307, USA; shallu1.kutlehria@famu.edu (S.K.); mandip.sachdeva@famu.edu (M.S.); 4 Graduate Program in Immunology & Microbiology, Baylor College of Medicine, Houston, TX 77030, USA; andrea.lee@bcm.edu

**Keywords:** aging, ocular surface, lacrimal gland, rapamycin, goblet cell, dry eye, inflammation

## Abstract

Dry eye disease (DED), one of the most prevalent conditions among the elderly, is a chronic inflammatory disorder that disrupts tear film stability and causes ocular surface damage. Aged C57BL/6J mice spontaneously develop DED. Rapamycin is a potent immunosuppressant that prolongs the lifespan of several species. Here, we compared the effects of daily instillation of eyedrops containing rapamycin or empty micelles for three months on the aged mice. Tear cytokine/chemokine profile showed a pronounced increase in vascular endothelial cell growth factor-A (VEGF-A) and a trend towards decreased concentration of Interferon gamma (IFN)-γ in rapamycin-treated groups. A significant decrease in inflammatory markers in the lacrimal gland was also evident (*IFN-γ*, *IL-12*, *CIITA* and *Ctss*); this was accompanied by slightly diminished *Unc-51 Like Autophagy Activating Kinase 1* (*ULK1*) transcripts. In the lacrimal gland and draining lymph nodes, we also observed a significant increase in the CD45^+^CD4^+^Foxp3^+^ cells in the rapamycin-treated mice. More importantly, rapamycin eyedrops increased conjunctival goblet cell density and area compared to the empty micelles. Taken together, evidence from these studies indicates that topical rapamycin has therapeutic efficacy for age-associated ocular surface inflammation and goblet cell loss and opens the venue for new investigations on its role in the aging process of the eye.

## 1. Introduction

The aging process induces changes in virtually all the tissues and organs from the body, and the eye is not an exception. Aging alters several ocular surface parameters, especially in women [1]. Age-associated changes in human tear composition, with an increase in inflammatory and remodeling factors, have been observed [2].

One of the mechanisms mediating age-related organ dysfunction is immunosenescence. This occurs due to changes in the immune cell phenotype, their activation state, and sensitivity to regulation, making the body more prone to autoimmune inflammation [3]. A common immune-mediated disorder of the ocular surface, especially in the elderly, is dry eye disease (DED) [4]. This condition presents with loss in the tear film stability, and can lead to the exposure of self-antigens and the breakdown in the mucosal tolerance. Tear film imbalance induces the maturation of resident antigen-presenting cells that present ocular surface antigens to T cells in the draining lymph nodes, which prime naïve CD4^+^ T cells. They differentiate into the cytokine-producing T helper (Th)-1 and Th-17 effector cells that cause ocular surface inflammation and epithelial disease [5,6,7,8]. Our research has discovered increased numbers of effector T regulatory cells in autoimmune mice, with rather impaired suppressive function, and accompanying age-related inflammation in the lacrimal gland [9]. Aged-associated murine dry eye presented with corneal barrier disruption, corneal surface irregularity, loss of conjunctival goblet cells, increased frequency of pathogenic T cells and increased production of cytokines and chemokines by cells in the ocular surface and lacrimal gland, mimicking the disease observed in humans [10,11,12,13]. De Silva also reported altered ocular surface parameters in mice in the course of aging, although they did not observe increases in the corneal dendritic cell density and tear osmolarity, findings normally associated with DED in humans [14].

Rapamycin is a natural macrolide, first isolated from plant and soil samples in the Polynesian islands, almost six decades ago. At the molecular level, rapamycin inhibits mammalian target of rapamycin complex 1 (mTORC1), which participates in key mechanisms of cell homeostasis, including autophagy and cell cycle progression. mTORC2 is another target of rapamycin, a molecule involved in the insulin signaling cascade [15]. This compound was first known by its antifungal properties, but rapidly, its efficacy against several immune-mediated diseases and for the prevention of allograft rejection became evident [16,17,18,19]. Later, the anti-aging effects of this molecule started to flourish. Rapamycin therapy extends the lifespan of several species of animals, enhances the generation of stem cells, and is neuroprotective [20,21,22,23]. In the eye, oral rapamycin has shown beneficial effects in preventing age-related macular degeneration in rats [24] or immune reactions after keratoplasty in humans [25,26]. On primary human corneal epithelial cells in vitro, rapamycin prolongs cell survival and decreases the proliferation, apoptosis, and expression of senescent markers. The treated cells showed less induction of inflammatory genes upon exposure to a Toll-like receptor (TLR)3 ligand [27]. However, the side effects of the systemic rapamycin administration, including thrombocytopenia, altered glucose homeostasis, and gonadal atrophy and increased breast tumor burden, limit its expanded use [28,29]. A dose-dependent exacerbation in autoimmune experimental uveitis was also observed after daily intraperitoneal administration of rapamycin in mice [30]. An option to avoid the toxicity of systemic drug delivery is the design of rapamycin formulations for local administration. Low-dose rapamycin-loaded polymer implants injected in close proximity to inter-species rat hind limb transplants improve graft survival [31]. Thapa and coworkers proposed a rapamycin delivery system using anti-CD9 monoclonal antibody-calcium carbonate nanoparticles to target senescent cells. The nanoparticles were wrapped in lactose-polyethylene glycol, and they showed efficient anti-senescent effects in old human dermal fibroblasts [32]. In a rat model of benign prostatic hyperplasia, Rapatar, a micellar nanoformulation of rapamycin, administered by oral gavage, improved hyperplastic histological changes in the prostate and decreased leukocyte infiltration in the stroma [33]. For the eye, Zhang and colleagues engineered rapamycin-loaded nanoparticles for local instillation during cornea transplantation [34].

Most importantly, in the non-obese diabetic (NOD) murine model of Sjögren syndrome, encapsulated rapamycin, either used systemically or in eyedrops, decreased lymphocytic infiltration of the lacrimal glands and improved cornea barrier disruption and tear volume [35,36]. This correlated with a decrease in some inflammatory markers. Subconjunctival injection of rapamycin microspheres yielded similar results [37]. However, the effects of topical Rapamycin in non-autoimmune, aged C57BL/6 mice, have not yet been evaluated.

Since aged C57BL/6 mice offer a great opportunity to investigate the efficacy of eyedrops in mice that spontaneously develop DED, the aim of our study was to ascertain the effects of the daily administration of encapsulated rapamycin eyedrops on the age-altered ocular surface phenotype. We found that our formulation reduces several inflammatory markers in the lacrimal gland after administration for three months. This was associated with increased percentages of CD4^+^Foxp3^+^ cells in the lacrimal gland and the draining lymphoid tissue. More importantly, mice exhibited an improvement in the goblet cell counts and area in the conjunctiva after the daily instillation of rapamycin. These results justify further investigations into the mechanisms underlying the protective effect of this natural compound in the immune-mediated ocular surface inflammation, to explore new drug formulations with improved efficacy and optimal delivery.

## 2. Results

### 2.1. Rapamycin Eyedrops Can Decrease the Concentration of Effector Lymphocyte Cytokines and Angiogenesis Mediators in Tears of Aged Mice

Rapamycin has been widely used as an anti-inflammatory and immunosuppressant in several animal models of autoimmune disease (including Sjögren Syndrome) [36] as well anti-aging studies to increase lifespan [38]. Rapamycin inhibits mTORC1 and mTORC2, which are master regulators of metabolism. On the other hand, our own group has demonstrated that aging in the mouse eye is associated with inflammation in the lacrimal gland and increased dry eye disease [9,10,11,13].

**Figure 1:**
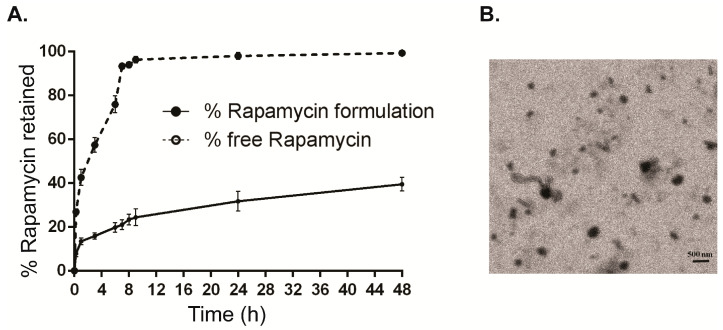
(**A**) Release study of rapamycin formulation for 48 h. (**B**) Transmission electron microscopy image of encapsulated Rapamycin (Black dots).

Rapamycin eyedrops were prepared following previous publication [36]. The particle size, zeta potential, and PDI of rapamycin formulation were 140.5 ± 4.5 nm, 2.3 ± 0.1 mV, and 0.3 ± 0.1, respectively. The encapsulation efficiency was 68.4 ± 0.4%, and drug loading was 1.21 mg/mL ± 0.07. Osmolality was 298 ± 2 mOsmol/kg. Furthermore, stability data demonstrated no significant change in the encapsulation efficiency over 1 month at −20 °C. Release studies revealed that 16.5 ± 1.4% of rapamycin was released from the eye drops in the first 3 h, as compared to 57.4 ± 3.3% drug release in the case of free drug. In addition, the formulation showed a sustained release effect, since 31.8 ± 4.5% was delivered over 12 h (Figure 1A). By TEM, the analysis particle was 130 ± 8 nm, which was comparable to that of DLS data (Figure 1B).

**Figure 2:**
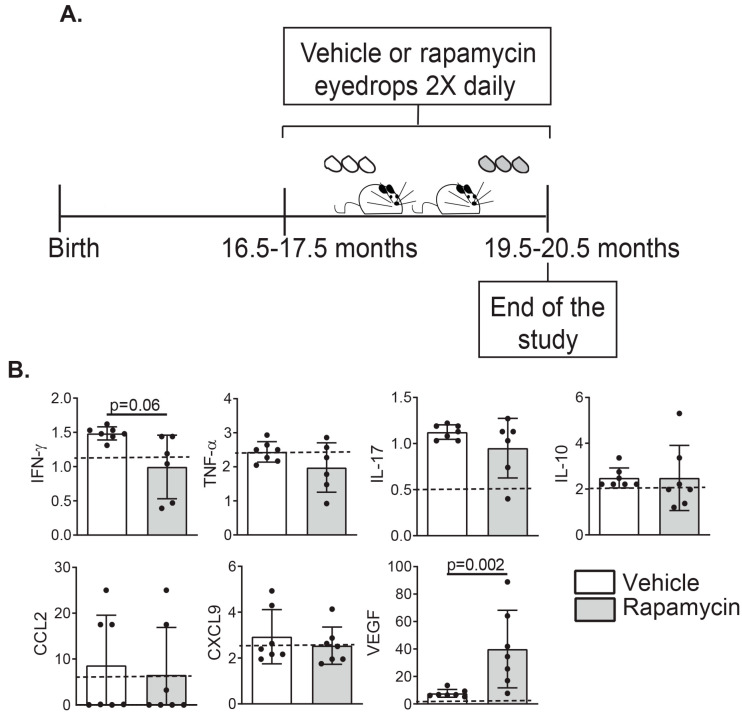
Schematic of the experimental set-up and immunological profile in the tears of the aged mice, and changes after treatment with Rapamycin eyedrops. (**A**) C57BL/6 mice aged about 17 months were instilled either with empty micelles or rapamycin BID for three months, when they were harvested to collect the tissues for further evaluation. (**B**) Tear washings were collected from the aged mice at harvesting and the levels of pro-inflammatory cytokines, chemokines, and VEGF-A in tears were evaluated using multiplex Luminex (*n* = 6–7/group, error bars indicate the standard deviation (SD) of the mean, Mann–Whitney test). The discontinuous line indicates the detection limit of the assay.

After successful fabrication, we tested the effects of daily topical administration on the ocular surface of either Rapamycin or its vehicle BID for three consecutive months in 17-month-old C57BL/6 mice (Figure 2A). First, we evaluated the levels of some of the pro-inflammatory cytokines in tears (Interferon (IFN)-γ, Tumor Necrosis Factor (TNF)-α, Interleukin (IL)-17, IL-10) that have been associated with DED [8,11,39,40]. We also include C-C motif chemokine ligand 2 (CCL2) and chemokine (C-X-C motif) ligand 9 (CXCL9) as markers of leukocyte chemotaxis and the vascular endothelial growth factor (VEGF-A), a signaling protein implicated in angiogenesis, induced by rapamycin [41]. A dramatic increase in the levels of VEGF-A in tears was evident in the Rapamycin-treated group (Figure 2B). We also observed that tears from rapamycin-instilled mice had a tendency towards decreased levels of IFN-γ compared to the vehicle-treated group. Moreover, a trend towards lower levels of other pro-inflammatory cytokines, such as TFN-α and IL-17 was noted, with no changes in the levels of IL-10 and other cytokines included in the multiplex array.

These data indicate that long-term topical instillation of rapamycin in the eye improves age-related ocular surface inflammation and prompted us to investigate the infiltration of immune cells in the lacrimal gland.

### 2.2. Rapamycin Eyedrops Decrease Pro-Inflammatory Markers in the Lacrimal Gland of Aged Mice with Minor Impact in Lymphocyte Infiltration and Autophagy

**Figure 3:**
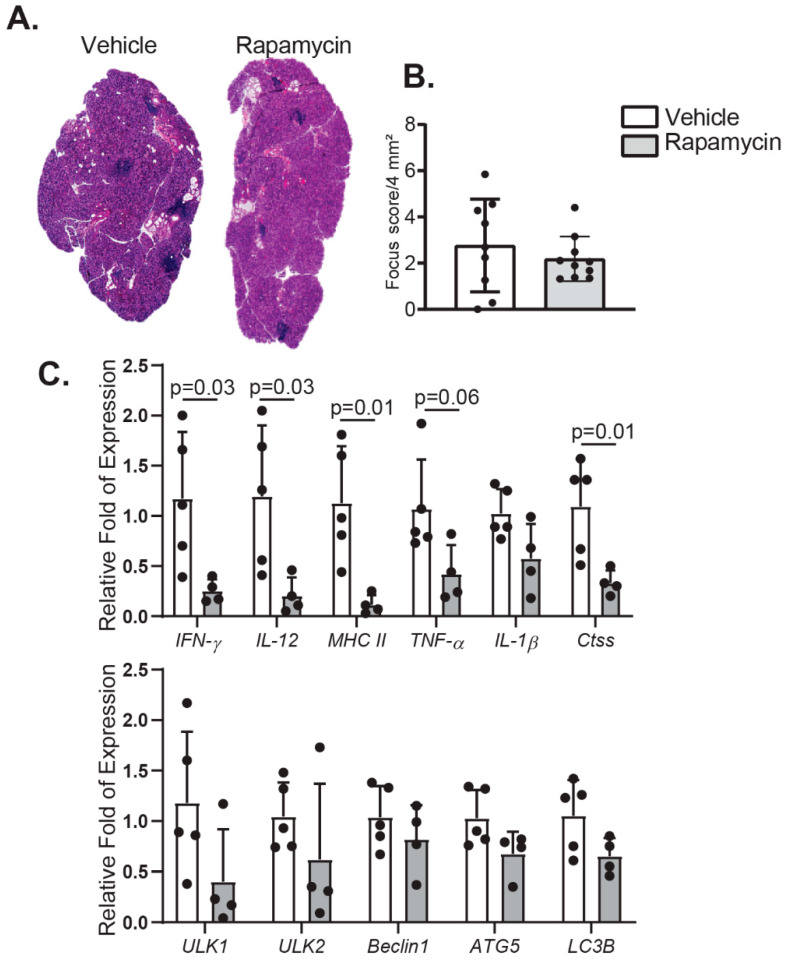
Degree of inflammation and autophagy in the lacrimal gland of the aged mice treated with rapamycin eyedrops. (**A**) Representative images of H&E stained histologic sections of lacrimal gland from aged mice instilled either with vehicle or rapamycin eyedrops, used to generate the graphs on B. (**B**) Focus score in the lacrimal gland was also calculated (*n* = 9–10/group, Mann–Whitney test). (**C**) Relative mRNA fold expression of either pro-inflammatory or autophagy markers in lacrimal glands were quantified by qPCR (*n* = 4–5/group, each dot represents one animal; error bars indicate the standard deviation (SD) of the mean, Mann–Whitney test).

Lacrimal glands undergo structural changes and dysfunction with aging [12,42]. One of the most evident changes is the increasing infiltration of immune cells in the gland [10,43]. To investigate the impact of rapamycin eyedrops in the eye of aged mice, we excised the extra-orbital lacrimal gland after treatment in both groups and processed for histologic evaluation and gene expression analysis. After Hematoxylin and Eosin staining, we counted the total amount of lymphocytic infiltration in these tissues. Although we found no significant decrease in the focus score in either vehicle- or rapamycin-treated mice, a tendency towards less inflammation was observed (Figure 3A,B). This was accompanied by significantly decreased levels of inflammatory marker transcripts, such as *IFN-γ*, *IL-12*, *MHC II*, and *Cathepsin S* (*Ctss)* in this tissue after rapamycin treatment (Figure 3C). An evident, although not significant, decrease in the level of *TNF-*α and *IL-1β* mRNA was also observed.

Rapamycin also activates the autophagy machinery through inhibition of mTORC1, which stimulates the formation of the Unc-51 Like Autophagy Activating Kinase (ULK)1-containing pro-autophagic complex and ultimately entails the formation of autophagosomes [44]. Therefore, we evaluate the mRNA messenger levels of several proteins involved in the formation of the autophagy–initiation complex. Surprisingly, most transcripts of the autophagy markers remain unchanged after instillation of rapamycin eyedrops in aged mice, although a mild decrease in *ULK1* mRNA was observed.

These data indicated that rapamycin eyedrops exert immunosuppressive effects in the ocular surface with a decrease in pro-inflammatory markers, not only in tears but also in the lacrimal gland.

### 2.3. Rapamycin Eyedrops Skewed the Effector Adaptive Immune Response Phenotype in the Aged Eye

**Figure 4:**
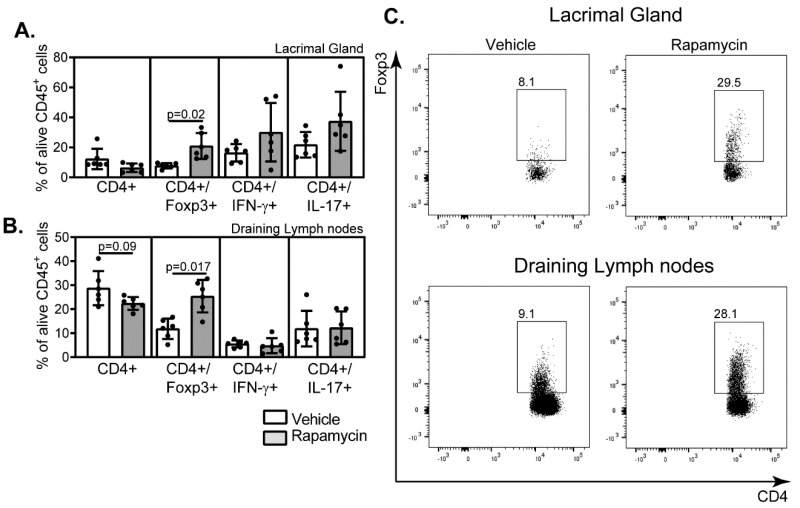
Effector immune cells in either the lacrimal gland or the draining lymph nodes of the rapamycin-treated aged mice. Either lacrimal glands (**A**) or the draining lymph nodes (**B**) from mice instilled BID either with vehicle or rapamycin eyedrops for three months were collected, and single-cell suspensions were obtained, to then subject them to stimulation with PMA + Ionomycin in the presence of a protein transport inhibitor as indicated in Materials and Methods. Then, cells were extracellular and intracellularly stained with fluorescent-antibodies as indicated. After gating out the death cells labeled with a fluorescent reactive dye, CD45^+^ leukocytes were selected, and among them, percentages of CD4^+^ cells expressing either Foxp3, IFN-γ or IL-17A were determined. (**C**) Representative dot plots of the percentages of CD4^+^Foxp3^+^ cells in the vehicle- or the rapamycin-treated mice in either the lacrimal gland or draining lymph nodes are shown. Squares within the dot plots enclosed the CD4^+^Foxp3^+^ subpopulation, and the numbers in the left corner indicate the percentages (*n* = 6/group, in (**A**,**B**), with each dot representing one animal; error bars indicate the standard deviation (SD) of the mean, Mann–Whitney test).

Previous reports from others and our own research have established that the impairment in the natural mechanisms of eye lubrication during aging is associated with greater infiltration of the ocular surface by effector immune cells [10,13,45]. Therefore, we evaluated the levels of CD4^+^Foxp3^+^, CD4^+^ IFN-γ^+,^ and CD4^+^ IL17^+^ leukocytes in the lacrimal gland and draining lymph nodes in the vehicle- or rapamycin-treated aged mice by flow cytometry. Interestingly, the long-term instillation of rapamycin as eyedrops changed the pattern of effector immune cells in the lacrimal gland and lymphoid-associated tissues towards greater percentages of CD4^+^Foxp3^+^ cells (Figure 4). A tendency towards decreased total levels of CD4^+^ cells was also noted in the draining lymph nodes.

These data indicate that local instillation of rapamycin can increase the percentages of CD4^+^Foxp3^+^ cells in the ocular surface during aging, potentially protecting the ocular surface from age-associated changes.

### 2.4. Goblet Cell Density Increased in the Conjunctiva of Rapamycin-Treated Aged Mice

**Figure 5:**
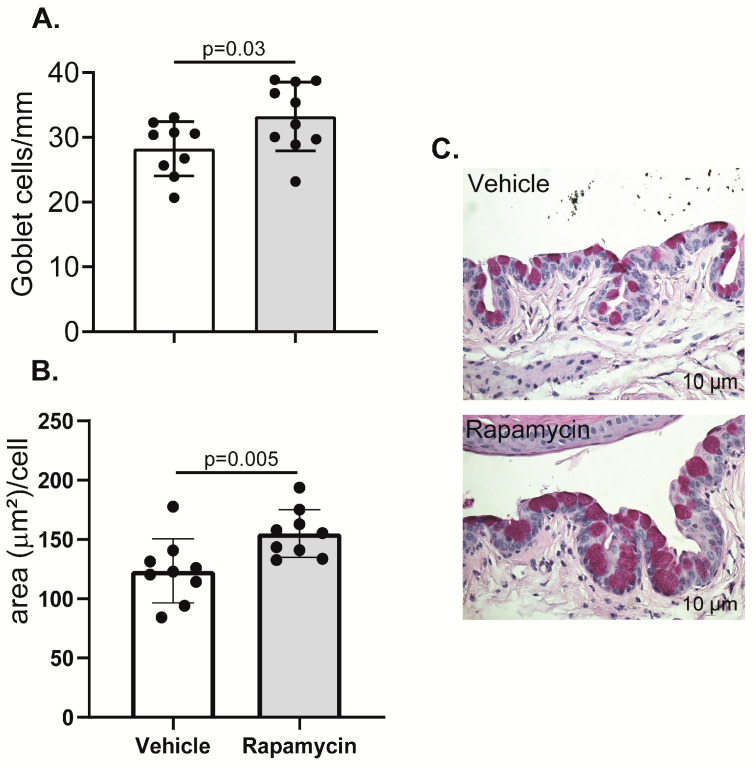
Rapamycin treatment rescues conjunctival goblet cells in aged mice. Eyes from the aged mice in each group were collected, fixed and embedded in paraffin, for histological processing and Periodic acid-Schiff staining. Finally, numbers of goblet cells in the palpebral conjunctiva of the whole eye were counted (**A**) and area was quantified (**B**) by light microscopy. (**C**) Representative image of the PAS+ goblet cells (purple) in the conjunctiva of either vehicle or rapamycin-treated mice (*n* = 9–10/group; each dot represents one animal and error bars indicate the standard deviation (SD) of the mean, Mann–Whitney test).

One of the most prominent changes in the ocular surface during aging is the progressive loss of goblet cells in the conjunctiva [10,11], that secrete mucins and immunomodulatory factors [46,47] further contributing to the lubrication and homeostasis of the eye. This is related to increased expression of pro-inflammatory cytokines, particularly IFN-γ, which is directly involved in goblet cell loss during desiccation and in aging [7,11]. Since we observed immunosuppressive effects of daily topical instillation of rapamycin in the eye, we proceeded to quantify goblet cell numbers in the upper and lower conjunctiva in aged mice treated with either vehicle or rapamycin eyedrops for the prior three months. Goblet cell density was chosen as an efficacy parameter because of the responsiveness of these cells to an inflammatory milieu [48,49]. We observed that rapamycin instillation significantly prevented the aging-related goblet cell loss in the conjunctiva. Moreover, goblet cells were not only increased in number, but they also showed more mucin content, as evaluated by the cell area in um2 (Figure 5).

## 3. Discussion

The present study demonstrates that rapamycin eyedrops are a potential therapy to suppress the deleterious effects of aging on the ocular surface and tear-producing tissues. Our experimental evidence shows a decrease in several pro-inflammatory cytokines in tears at the protein level and in the lacrimal gland at mRNA level, together with an increase in the percentages of T regulatory cells (CD4^+^Foxp3^+^) in the lacrimal gland and draining lymphoid tissue in the aged mice treated for three months with the encapsulated rapamycin. We also observed a tendency towards lower percentages of CD4^+^ T cells in these tissues after long-term rapamycin exposure. These results are consistent with a previously published study reporting lower levels of IL-1β in the brain of aged mice fed long-term with rapamycin, which also correlated with an improvement in learning and memory [50]. Shat et al. also observed decreased transcripts of *IFN-γ, IL-12A, TNF-α and MHC class II* in the lacrimal gland after rapamycin instillation on the eye [36]. Similar to our results, they also observed decreased Cathepsin S activity in tears and lacrimal gland lysates together with reduced expression of the *Ctss* gene, encoding Cathepsin S, in the lacrimal gland [36]. Since a previous report from the same group has demonstrated increasing *Ctss* expression and activity in the NOD mouse model of autoimmune dacryoadenitis, mainly produced by infiltrating macrophages [51], these results further support the anti-inflammatory effects of rapamycin.

The ability of rapamycin to increase the levels of CD4^+^Foxp3^+^ T cells has also been demonstrated in other experimental models and humans [52,53,54]. Kopf et al. also showed a concomitant dose-dependent decrease in the levels of IL-17^+^ cells in cultured mouse CD4^+^ T cells after activation in vitro [52]. In our experiments, we did not observe an association between the increase in CD4^+^Foxp3^+^ cells and the percentages of other effector T cell subpopulations (either CD4^+^IFNγ^+^ or CD4^+^IL-17A^+^) in the lacrimal gland or draining lymph nodes. Basu et al. proposed a mechanism by which Foxp3^+^ T regulatory cells are resistant to the immunosuppressive effects of rapamycin. They demonstrate that Foxp3 induces the expression of Proviral Integrations of Moloney virus 2 (Pim2), a serine/threonine kinase that confers T regulatory cells a growth advantage in the presence of rapamycin [53]. Moreover, bone-marrow-derived dendritic cells generated in vitro in the presence of rapamycin preferentially promote the proliferation of T regulatory cells expressing Foxp3 [55].

More importantly, our data showed an improvement in goblet cell numbers and area in the conjunctiva after the long-term rapamycin instillation. Systemically administered rapamycin reduces Muc5ac and CLCA3 in the airways in a mouse model of asthma, [56]. However, this study also showed reduced levels of activated CD4^+^ T cells and CD25^+^Foxp3^+^ regulatory cells in the lungs of rapamycin-treated mice. Tissue-specific differences in immune regulation might account for these discrepancies in the two disease models (an increase in goblet cells is beneficial for dry eye while it is pathogenic in asthma models). Interestingly, when mTOR genes are conditionally disrupted in mice, a reduction in the load of Muc2 in goblet cells is observed, with no reduction in the number of goblet cells in the intestine [57]. More studies need to ascertain the influence of Rapamycin and the mTORC regulators in the differentiation and homeostasis of goblet cells in the different mucosal tissues. On the other hand, other functional tests to evaluate DED in mice might have also shed light on the benefits of the Rapamycin eyedrops during aging. However, our own work [10] has demonstrated that aged mice have not only signs of dacryoadenitis but also corneal irregularities, starting at 6–9 months of age. Although we have tried to measure corneal barrier function in aged mice at 24 months of age by using the fluorescent molecule Oregon-green-dextran (OGD), the frequent presence of auto-fluorescent cataracts in these mice hinders accurate measurement of this parameter. Moreover, we and others [10,14,58] have shown that tear secretion significantly increases in apparently healthy C57BL/6 female mice with aging and, therefore, increased basal tear flow cannot be used as a read-out to show improvement in the ocular surface lubrication after long-term application of Rapamycin eyedrops. Moreover, De Silva and colleagues also showed, for example, that aged C57BL/6 mice have lower corneal sensitivity using the Cochet–Bonnet esthesiometry [14]. They also used tomography to evaluate corneal thickness or a combination of immunofluorescence and confocal microscopy to evaluate the density of nerve projections and dendritic cells in the ocular surface. In another model [58], the trigeminal ganglion branches and axonal branches, which support basal tearing and blinking, were examined and are reported to change drastically in aged mice. It would be interesting to ascertain if Rapamycin eyedrops could improve these parameters with aging, beyond the effects we observed as immune modulator.

Rapamycin therapy in our experimental design failed to diminish lymphocytic infiltration, visualized by histology, in the aged lacrimal gland, despite the decreased levels of pro-inflammatory cytokines and increased percentages of CD4^+^Foxp3^+^ cells observed in this organ after the therapy. However, inhibiting the lacrimal gland T cell infiltration during aging might prove more challenging, as Harpaz et al. demonstrate that aged effector T cells are refractory to the suppression by T regulatory cells, then perpetuating ocular inflammation [59]. It is also possible that treatment with Rapamycin for a longer period than 3 months might be beneficial and warrants further investigation.

Among the autophagy markers, only *ULK1* was slightly responsive to rapamycin eyedrop administration in the present study. In other reports, the effects of rapamycin, used either systemically or locally, on levels of autophagy markers in lacrimal glands is rather variable [35,36]. However, the pro-autophagic effects of rapamycin are mainly regulated in a post-transcriptional manner, and we only investigated mRNA levels. For example, the conversion of the cytoplasmic Microtubule-associated protein 1A/1B-light chain (LC3)-I to the autophagosome-bound LC3-II is one of the indicators of the autophagic flux in cells and tissues [60]. This was demonstrated by Choo B-J in the lacrimal gland, after administering rapamycin systemically [61]. On the other hand, mTORC1-independent effects of rapamycin have also been reported elsewhere [15,62].

Interestingly, we observed a significant upregulation in the protein levels of VEGF-A in the tears of the rapamycin-treated mice in our model. This finding conflicts with those obtained in the model of endoplasmic reticulum stress-induced DED [61]. In that study, Choo B-J demonstrated that systemically administered rapamycin decreases cornea barrier disruption and lacrimal gland hypoxia by decreasing levels of angiogenic factors, such as VEGF-A. They argue that vascular endothelial cell proliferation might be detrimental to the lacrimal gland [61]. Although we do not know the reasons for this discrepancy, VEGF-A regenerates nerve injury in the cornea, which might be beneficial in DED [63,64].

We envisage that new formulations of rapamycin will improve its efficacy for the treatment of DED. Lee et al. proposed humanized bi-headed polypeptides for the subcutaneous delivery of rapamycin [65]. They applied this delivery system in the NOD mouse model of autoimmune dacryoadenitis, obtaining a beneficial reduction in lymphocytic infiltration in the lacrimal gland and levels of some pro-inflammatory cytokines in this tissue with no systemic toxicity. The same group also designed an ICAM-1 targeted protein-polymer carrier for rapamycin that specifically binds to ICAM-1 with potential use for inflammatory conditions such as DED [66]. Nutraceutical compounds mimicking the activity of rapamycin, and other mTORC1 inhibitors are also being screened [67,68].

Taken together, our data demonstrate that long-term therapy with rapamycin eyedrops could potentially improve tear film stability in the aged ocular surface. The potential mechanism mediating these effects is likely related to the ability of rapamycin to decrease inflammation and increase the recruitment or generation of T regulatory cells to lacrimal gland and surrounding lymphoid tissue. Whether rapamycin also revitalizes these cells, improving their suppressive function, warrants further research. New strategies for drug design and delivery are also needed to prevent undesirable side effects of this compound while optimizing its tear-regenerating activity in the aged eye.

## 4. Materials and Methods

### 4.1. Reagents

Rapamycin (LC Laboratories, Woburn, MA, USA), 1, 2-distearoyl-*sn*-glycero-3-phosphoethanolamine-*N*-[methoxy (polyethylene glycol)-2000] (DSPE-PEG2000) was manufactured in Lipoid LLC (Newark, NJ, USA). Chloroform, methanol and Dulbecco’s Phosphate Buffered Saline (DPBS) were purchased from Sigma Aldrich (St. Louis, MO, USA).

### 4.2. Preparation and Characterization of Rapamycin Eyedrop Formulation

Rapamycin was dissolved in methanol and DSPE-PEG2000 in chloroform in a ratio of 1:1. The solvents were evaporated using the BUCHI Rotavapor R124 (BÜCHI Labortechnik AG, Flawil, Switzerland), leaving behind a thin film which was then solubilized in DPBS. The formulation was then centrifuged at 10,000 rpm for 10 min to remove un-encapsulated rapamycin. The formulation was filtered through a 0.22 µm PES syringe filter (VWR, Radnor, PA, USA) for sterilization. The control micelles were prepared similarly in the absence of the drug.

The hydrodynamic diameter, zeta potential, and polydispersity index (PDI) of the nanoparticles were determined in a Nicomp 380 ZLS (Santa Barbara, CA, USA). Transmission electron microscopy (TEM) studies were carried out in a Philips/FEI Biotwin CM120 (Eindhoven, The Netherlands) at 120 kV using a formavar/carbon-coated copper grid (Electron Microscopy Sciences, Radnor, PA, USA), negatively stained with 2% ammonium molybdate (Sigma Aldrich, St. Louis, MO, USA). High-performance liquid chromatography (HPLC) was used to determine drug assay, the drug encapsulation efficiency and loading. After preparation, drug assay was performed to determine total amount of drug. The formulation was then centrifuged at 10,000 rpm for 10 min. The supernatant was collected, diluted with the mobile phase, and analyzed by HPLC. Drug encapsulation efficiency (EE) was calculated as drug content obtained after centrifugation × 100/assay content of the formulation. Drug loading was determined as the amount of drug loaded per ml of the formulation. The concentration of rapamycin was determined by the normal phase HPLC column (Lichrosorb 5 SIL 60A, Phenomenex, Torrance, CA, USA) (4.6 × 250 mm^2^, 5 µm) at 278 nm wavelength. The mobile phase was acetonitrile: methanol: water (20:60:20 *v*/*v*/*v*) used at a constant flow rate of (0.5 µL/min) and detected using Waters 2996 photodiode array detector. Osmolality was calculated using Osmomat 3000 basic (Gonotech GmBH, Berlin, Germany). The formulations were dispensed in small aliquots and stored at −20 °C for up to one month when the remaining formulations were discarded. A fresh batch of encapsulated rapamycin and the empty vehicle was prepared every month.

### 4.3. Rapamycin Release Studies

The rapamycin release was determined by using a dialysis bag technique in DPBS (pH 7.4) under sink conditions at room temperature. Rapamycin formulation (1 mL) was loaded in a dialysis bag (MWCO of 10,000 Da; Sigma-Aldrich) and immersed in 20 mL of DPBS. As a control, 1.8 mg/mL of rapamycin was dispersed in 3% DMSO, loaded in a dialysis bag, and samples were collected at appropriate time intervals. The amount of rapamycin released was determined by using HPLC, as described previously.

### 4.4. Animals

All animal experiments were approved by the Institutional Animal Care and Use Committees at Baylor College of Medicine (protocol #7342, first approved 3 March 2017). All studies fulfilled the requirements of the Association for Research in Vision and Ophthalmology for the Use of Animals in Ophthalmic and Vision Research and to the National Institutes of Health Guide for the Care and use of Laboratory Animals [69].

The experiments were performed at the Ocular Surface Center, Department of Ophthalmology, Baylor College of Medicine, Houston, Texas.

Aged female C57BL/6J mice (The Jackson Laboratory, Bar Harbor, ME, USA), were maintained in specific-pathogen-free vivarium and were used at 16.5–17.5 months (*n* = 30).

Mice were housed at pathogen-free facilities of Baylor College of Medicine and were kept on diurnal cycles of 12 h/light and 12 h/dark with ad libitum access to food and water.

Because dry eye is more frequent in women [4], and aged male mice do not develop corneal barrier disruption (a hallmark of dry eye) [11], only female mice were used.

### 4.5. Rapamycin or Vehicle Eyedrops Dosing Regime

Mice were randomized to receive bilateral topical treatment with either Rapamycin or Vehicle eye drops (5 uL/eye, BID) for 3 continuous months, including weekends. After 3 months, mice were euthanized by isoflurane inhalation followed by cervical dislocation. Tissues were collected and processed. Efforts were made to collect multiple tissues from the same mouse whenever possible. An exact sample size per test is displayed in the figure legends.

### 4.6. Tear Washings and Multiplex Cytokine Immunobead Assay

Tear washings were collected from live mice one week before the end of the treatment as previously described [70]. Using a manual pipette, a 1.5 μL of PBS + 0.1% BSA eye drop was instilled on the ocular surface. Using a 1-μL volume glass capillary tube (Drummond Scientific Co., Broomhall, PA, USA), the tear washings were collected at the tear meniscus in the lateral canthus taking care not to touch the conjunctiva and initiate reflex tears. Tear washings from four eyes were pooled into one sample (four eyes/4 μL) in PBS + 0.1% bovine serum albumin (BSA) (6 μL), centrifuged briefly and snap frozen in liquid nitrogen. Samples were stored at −80 °C until the assay was performed. There were 6–7 samples per group.

To detect IFN-γ, TNF-α, IL-17, IL-10, CCL2, CXCL9, VEGF-A, among others, mouse monoclonal antibodies coupled to beads (MCYTOMAG-70K, Millipore, Burlington, MA, USA) were used as previously reported and the reactions were detected with streptavidin-phycoerythrin using a Luminex 100 IS 2.3 system (Austin, TX, USA) [49]. The lower limits of this assay were 1.1 pg/mL for IFN-γ, 2.3 pg/mL for TNF-α, 0.5 pg/mL for IL-17, 2.0 pg/mL for IL-10, 6.7 pg/mL for CCL2, 2.4 pg/mL for CXCL9 and 0.3 pg/mL for VEGF-A.

### 4.7. RNA Isolation and Real-Time PCR

Total RNA from lacrimal glands was extracted using a QIAGEN RNeasy Plus Mini RNA isolation kit (Qiagen, Hilden, Germany) following the manufacturer’s instructions. RNA concentration was measured and 1 ug of total RNA was used to synthesize cDNA (Ready-To-Go™ You-Prime First-Strand kit, GE Healthcare, Chicago, IL, USA). Real-time PCR was performed using specific MGB Taqman probes for IFN-γ (*Ifng*, Mm01168134), IL-12 (*il12a*, Mm00434165), major histocompatibility complex class II (*CIITA*, Mm00482914), TNF-α (*TNF*, Mm00443258), IL-1β (*Il1b*, Mm00434228), Cathepsin S (*CTSS*, Mm 01255859), Unc-51 like autophagy activating kinase (ULK)1 (*ULK1*, Mm00437238), ULK2 (*ULK2*, Mm03048846), Beclin1 (*BECN1*, Mm01265461), Autophagy related (ATG)5 (*ATG5*, Mm01187303), Microtubule-associated protein 1B-light chain (LC3)b (*MAP1LC3B*, Mm00782868) using a Taqman Universal PCR system (StepOnePlus™ Real-Time PCR System, Applied Biosystems, Bedford, MA, USA). The hypoxanthine phosphoribosyltransferase 1 (*HPRT1*, Mm00446968) gene was used as a housekeeping gene for each reaction. Ct values were then normalized by HPRT values [71].

### 4.8. Histology, PAS Staining, and Quantification of Focus Score in Lacrimal Glands

Eyes and ocular adnexa were surgically excised, fixed in 10% formalin for at least 24 h, and later processed and paraffin embedded. Then, 5-µm sections were cut using a microtome (Microm HM 340E, ThermoScientific Wilmington, DE, USA). Whole-eyeball sections were cut and stained with Periodic Acid Schiff (PAS) reagent. The goblet cell density and area was measured in the superior, and inferior bulbar and tarsal conjunctiva using NIS-Elements software, BR, 64 bit 3.22.15 (https://www.nikonmetrology.com/en-gb/product/nis-elements-microscope-imaging-software) and expressed as the number of positive cells per millimeter [72] or area (µm^2^) per cell.

Focus scores were counted in haematoxylin and eosin-lacrimal gland sections by standard light microscopy using a 10X objective from an Olympus CX31 microscope (Tokyo, Japan). Foci composed of a minimum of 50 mononuclear cells were counted and recorded in a database. Slides were scanned to obtain digital images using PathScan Enabler V (Meyer Instruments, Houston, TX, USA). The area of the scanned lacrimal gland was then measured using NIS Elements software and focus score/4 mm^2^ was calculated. We evaluated three non-consecutive sections out of five possible non-consecutively. The number of foci per section was adjusted to the scanned area of that section and the results of the focus score per section were averaged among each animal. The final score is the average of all glands within the group. We used a blinded reviewer.

### 4.9. Flow Cytometry Analysis

Extra-orbital lacrimal glands and draining lymph nodes were excised and prepared, as previously described [73]. Single-cell suspensions from young and aged lacrimal glands were incubated with anti-CD16/32 (BD Biosciences, 4 °C, 10 min), and subsequently stained using CD45 (clone 30-F11, BioLegend, San Diego, CA, USA) and CD4 (clone Rm4-5, BD Biosciences, Bedford, MA, USA), followed by intracellular staining. To accomplish this, single-cell suspensions were incubated for five hours in the presence of 5% CO_2_ with 0.5 µL Golgi Stop™ (BD Biosciences), 0.5 µL Golgi Plug™ (BD Biosciences), PMA (0.5 µg) (Sigma-Aldrich, St. Louis, MO, USA), ionomycin (0.5 µg) (Sigma) in 1 mL in complete RPMI 1640. Cells were stained with an infra-red fluorescent viability dye (Life Technologies, Grand Island, NY, USA) before incubation with a Fixation/Permeabilization working solution (eBioscience, ThermoFisher Scientific, Waltham, MA, USA) for 18 h. This was followed by incubation with anti-IFN-γ_Pacific Blue (BioLegend, Clone XMG1.2), Foxp3_APC (eBioscience, clone FJK-16s) and anti-IL-17_PE (eBioscience, Clone 12-7177-81), as previously described [74]. Negative controls consisted of fluorescence minus one splenocytes.

The following gating strategy was used: lymphocytes were identified by forward, and side scatter properties and after including singlets and excluding dead cells, CD45^+^ cells were gated and followed by identification of CD4^+^ cells. Further gating was used to investigate the frequency of CD4^+^Foxp3^+^ or CD4^+^IFN-γ^+^ or CD4^+^IL-17^+^ cells. At least 100,000 events or more were acquired with a BD Canto II Benchtop cytometer (BD Biosciences). FlowJo software version 10 was used for data analysis (Tree Star Inc., Ashland, OR, USA).

### 4.10. Statistical Analyses

Statistical comparison between the two different groups, namely mice subjected to Rapamycin eyedrops or empty micelles, was performed using the Mann–Whitney U test. Differences were considered statistically significant when the *p* value was less than 0.05. Statistical analyses were performed using the GraphPad Prism software Version 8.4.1 (GraphPad Software Corporation, San Diego, CA, USA).

## Abbreviations

DED	Dry Eye Disease
Th	T helper
mTORC1	Mammalian target of rapamycin complex 1
NOD	Non-obese diabetic
TEM	Transmission electron microscopy
HPLC	High performance liquid chromatography
EE	Encapsulation Efficiency
IFN-γ	Interferon gamma
TNF-α	Tumor Necrosis Factor alpha
IL	Interleukin
CCL2	C-C motif chemokine ligand 2
CXCL9	Chemokine (C-X-C motif) ligand 9
VEGF-A	Vascular endothelial growth factor-A
ULK1	Unc-51 Like Autophagy Activating Kinase 1
PAS	Periodic Acid Schiff
LC3	Microtubule-associated protein 1A/1B-light chain 3
